# Bibliographical Notices

**Published:** 1846-11

**Authors:** 


### BIBLIOGRAPHICAL NOTICES.

*The Structure and Functions of the Female Breast, as they relate to its Health, Derangement and Disease.* By E. W. Tuson, F. R. S., F. L. S., Surgeon to the Middlesex Hospital 8vo. pp. 485. London: 1846.

This is a singular production from one holding so prominent a post as Surgeon to one of the London Hospitals; who is withal an F. R. S., and F. L. S., and who, it seems, has already appeared *six times*—

"Insatiate Tuson! could not *once* suffice"?—

before the public, and from whom, he says, he has already experienced "liberality." To the profession, however, he is not very complimentary, for they do not appear to uphold him in certain of his—what appear to us—empirical propositions.

"In introducing new remedies," he says, "many difficulties have presented themselves, which I must own could have been little anticipated, particularly amongst members who fancy themselves branches [?] of a liberal profession, who on many occasions have been pleased to term my endeavour to advance the practice of medicine 'Quackery,' for giving those remedies of which I knew not the dose or effects." p. xiii.

Most certainly does the charge of quackery appear to us to apply to him for many of his remedies proposed in the present volume; yet they are not all recommended without some vague hypothesis being stated as the rule of conduct, of which the following may be regarded as a specimen:

"We are indebted to Mulder for the discovery of proteine and its compounds; and Scherer analyzed this original matter prepared from animal albumen and fibrine, from the crystalline lens, from hair, and from horn, and the results of all these analyses agreed with the formula $C_{48} H_{36} N_6 O_{14}$, which is about identical with the blood in the healthy state. From a variety of practical observations, made during a period of nearly twenty-five years, a great portion of this time [?] having been devoted to the bed-side of the patient, I have been frequently struck with the want of power in the system to enable the constitution to bear up against disease [! !]; and as proteine entered into the formation of several of the tissues (being identical with blood) of the frame," [we do not see the *sequitur*,] "I was led to investigate its effects upon the system. I found it beneficial in repairing certain decayed structures," [of what kind ?] "in assisting the functions of nature in producing certain portions of the animal frame, which had become weakened from a want of solidity; and under various circumstances attendant with debility. Its use became serviceable. The preparation was made according to the directions given in Turner's chemistry, and the results will be seen in these pages. But since that time Baron Liebig's researches have tended to disprove the existence of proteine: we must therefore call it by some other name. It has been administered in numerous instances; and a something can be procured which has been beneficial, and will be so in future: therefore, until we have a fresh term for this original matter, we must be at present satisfied by calling it 'proteine.'" p. xv.

This is a sample of the author's therapeutical reasoning, and it is one of the best. He is evidently one of the chemical school of Liebig, but does not appear to possess much of the spirit of his great leader,—from whom, by the way, he extracts in a wholesale manner.

"Chemistry," he says, "has far exceeded in discoveries our practical knowledge of the effect of medicine; and if we consult any work on that subject, we must be struck with the number of new preparations that are described, and which have never been used in our practice: their actions are still unknown; so that the greatest benefits may hereafter result from the exhibition of many of these productions. Chemistry has enabled us to understand the composition of several parts of the animal frame; and in these pages I have had occasion to point out that certain parts of the body possess particular chemical properties. Thus the brain and spinal marrow have entering into their formation certain acids and products, which are not present in other structures, and which are perfectly distinct from other animal tissues. I [?] have pointed out that brain and nervous matter contain cerebric acid, oleo-phosphoric acid, cholesterine, &c. May not some diseases of the brain and nervous system depend upon a deficiency or an excess of one or more of these chemical constituents? When urea abounds in the system, we know the consequence—the brain becomes affected and the patient dies. May not the stomach cause a greater quantity of these acids to exist? and may not this be the cause of certain affections of the mind and nervous system?—or may not the stomach be deficient in supplying these chemical agents which may be essentially necessary to health? What effect would these products have upon the constitution, and upon certain diseases of the brain and nervous system? What effect would cerebric acid have in certain deranged states of the mind? What effect oleo-phosphoric acid? These are questions that I am not at present prepared to answer, but in a short time hope to be able to place before the public some interesting particulars on this and on other points." p. xi.

These "interesting particulars" we are satisfied, will never come; and the suggestions themselves can only be regarded as gratuitous. When the views of Liebig in regard to the constitution of the encephalic neurine were first published, we ventured to affirm publicly, that some wiseacre would be sure to ascribe disease of the encephalon to an excess or defect of one or more of the essential constituents; and it was not long before we heard of a physician prescribing phosphorus to a nervous lady on the vague supposition that the malady might be owing to a defect in the proportion of phosphorus;—as if disease did not always consist rather in mal-assimilation, than in deficiency of pabulum. From Mr. Tuson's researches, we confess we are amongst the "blessed" who expect nothing. Notwithstanding his pretensions, his acquaintance with chemical science is evidently by no means profound; and at times his chemical reasoning borders absolutely on the ridiculous.

The Author commences his work with an "anatomical examination into the structure and functions of the mammary glands," in which we are told sundry facts that are new to us:—for example, that "the period of *maturity* commences at puberty, and ends about the forty-fifth or fiftieth year." During the 'age of youth,' "the pelvic viscera and mammæ are rapidly developed; the hips become enlarged; the thoracic viscera *expand, increasing the mammæ*" [!]. Each *lobe* of the breast is composed or made up of several *lobes,* formed by rounded granulations of a rosy white colour, about the size of a poppy-seed. "It is supposed, that each of these granulations are [is] composed or formed by the union of a number of small vessels"[?]. "Around the nipple there is a circle of skin or disk." "In the want of ovaria, the breasts will remain, as in an early stage of life, undeveloped; but a false conception is attended with a fulness of the mammæ, so that *their* existence must have considerable influence upon *their* functions." "Any derangement in one or more of the organs necessary to be in health for the due *performance of the menstrual period* [!], will more or less affect the mammary glands, *the severity depending upon the primary cause.*"

Page upon page is here extracted from Liebig's "Organic Chemistry," to give a "short notice of the chemical characters of some of the tissues of the animal economy." In treating of lactation, Mr. Tuson informs us that "sometimes, when the child is brought to the mother, she feels an internal commotion of the breasts, *influenced, no doubt,* by the thoracic viscera." "The maternal office of suckling is always attended with a calm serenity of mind, *scarcely felt in other situations:* and the suppression of milk, on its first appearance, with irritability, languor, or despondence." "It is stated upon authority, that girls of the best character, by the irritation of a child sucking, have *become able to support it;*"—a consolation certainly for needy young women placed in such circumstances! "The number of teats or nipples in different animals corresponds usually to the number of their young: *they are generally even:* sometimes in women there is an additional nipple" [when they have twins, we presume] "or more, and sometimes these have been *discovered only by accidental causes.*" These extracts will sufficiently exhibit the author as an anatomist, a physiologist, and a writer. In the last respect, nothing assuredly could be worse.

Mr. Tuson next proceeds to classify the diseases of the breast; and the same confusion of intellect and inaccuracy of expression are exhibited here as in the previous sections. We have not space, however, to go into many specifications. Under "undue lactation," we are told that "the intermarriages of cousins have been fully established to produce weak and delicate children by

*the admixture of the blood of one branch*"—an assertion which we do not clearly comprehend. Speaking of milk abscess, he says, "An endeavour ought to be made to disperse these abscesses, *the same* as the treatment of another complaint"! In a case of this kind, Mr. Tuson prescribed the *hydrochloride* [hydrochlorate] *of ammonia!* Eighteen pages from a paper by Cruveilhier constitute the remarks on fibrous tumours; and Sir Benjamin Brodie and Dr. Copland minister largely to the articles on encysted tumours, and on cystic and hydatid tumours of the breast. To the class of organic lesions, comprising tumours or formations of a malignant and contaminating character, Mr. Tuson has devoted most of his attention. His desire manifestly is to be regarded by the public as an experienced "cancer doctor." It is strange, that the excellent article in the Cyclopædia of Surgery by Dr. Walshe, which was re-printed in a separate form in this country, should have wholly escaped his attention, and that he should state Müller to be "the latest and best authority" on the subject. In lieu of Müller, however, we have six pages containing Sir E. Home's views on the formation of scirhous tumours from Sir Astley Cooper's Lectures, and thirty-four consecutive pages from Dr. William Budd. Dr. Copland's Dictionary furnishes several pages to Fungus Hæmatodes.

We pass over Mr. Tuson's pseudo-scientific twaddle on the mode in which cancer is produced. Here, Turner and Bostock, Marcet, Prout, Liebig, Müller and others are laid under heavy contributions, often without our being able to discover the precise object of the author. We shall notice briefly, however, the armamentarium which he brings to bear on this formidable malady. We may remark, by the way, that he seems occasionally to take from others without the slightest indication of the authority; as, for example, when he says, in speaking of the treatment of cancer—"The section of Van Swieten, *which I mentioned to you* to-day on the subject of cancer, is 499." Mentioned to whom? This is marvellously like as if it were extracted from some published lecture.

*Chloride of zinc* comes in for favourable mention, and we are told that "*Dr. Conguoin* [Canquoin] in Paris" employed it. "But this remedy," he says, "like many of the preparations that have been recommended, is only of use in arresting the progress of this formidable disease. We are flattered by vain anticipations of curing the disorder, buoyed up with hope which is fallacious and disappointing, whilst the cancerous disease continues slowly in its progress, daily gaining an ascendancy upon the constitution, until death alone checks its malignant propensity." p. 401.

The effect produced by the internal administration of one-third or half-a-grain in water, even once a-day, "changed the character of the cancer from a diffused form, which extended over a large surface, to a more circumscribed extent." p. 409. The preparation, he says, affected the gums in the same way as preparations of mercury; and in one case, the medicine was obliged to be discontinued for this reason.

Solutions of *chloride of calcium* in water, Mr. Tuson affirms, will be found very useful in many foul, ulcerated cancerous affections, especially when the fœtor is excessive. *Chloride of carbon* is, however, most highly recommended by him. He gives it internally in the dose of one drop, at night, in water, and increases the dose to two or three drops. "In many cases it produced perfect freedom from pain, quieted the mind and nervous system generally, prevented the rapid growth and progress of the disease; and rendered the patient's life *comparatively happy to their previous feeling and condition.*" p. 412. In every form of cancer it proved an excellent anodyne, given internally, as well as applied locally. The usual strength, as a local application, is a drachm of the chloride to a pint of water. Mr. Tuson recommends it mixed with water as a gargle in foul ulcerated sore throats, and in various other affections where disinfectants are needed. The article used by him under the name *chloride of carbon* has been variously termed by modern chemists—*chloroform, chloric ether, terchloride of carbon, chloride of formyle,** &c. Mr. Tuson has seen a favourable change take place in cancerous ulcers from the local application of *Heuchera,* alum root; but it soon ceases to have the same effect. "In some cases when the cancer bleeds, it may be very advantageously applied, as it frequently prevents hemorrhage. p. 424.

* For its mode of preparation, medical properties, &c., see the Fifth Edition of Dunglison's New Remedies, p. 612. Philadelphia, 1846.

*Perchloride of copper*, formed by dissolving peroxide of copper in muriatic acid, and evaporating to dryness by a heat below 400°, has been used by him on many occasions to cancerous ulcerated sores, in the form of a lotion, made of the strength of six grains to an ounce of water. "I consider it likely," he concludes, "to be very beneficial *under certain circumstances*." p. 426.

Of *chloride of potassium* he speaks as follows:—"I have administered this preparation, internally, in doses of from three to six grains, twice and three times a-day: the patients have been unable to continue it on account of it [its] causing uneasiness and troublesome sensations in the head." p. 426.

Can such be the effect of the chlorohydrate or muriate of potassa? And what effect in cases of secondary eruption could be expected from four grains of this neutral salt dissolved in one ounce of water, which Mr. Tuson says is "one of the best lotions that can be prescribed"!

*Chloride of lead* was employed by him, "both in the form of lotion and ointment with some success."

Other remedies, commonly given in cancer receive a passing notice from Mr. Tuson; but the above are those which he advises; and most of all—as before remarked—the chloride of carbon. We confess, however, that we rise from the perusal of his statements and cases with, we think, well-founded doubts as to the accuracy of his observation, and the purely professional character of his motives. The whole work seems, indeed, as if it were intended to impress the public rather than the profession; and we feel compelled to say, that we have not seen, recently, any production from any source, respectable by position, which has given us a more unfavourable opinion of the professional and literary qualifications of its author. Yet notwithstanding this, we should not be surprised if it should be reprinted in this country; so great is the rage for re-producing every thing that appears abroad.

*Chemistry of the four seasons, Spring, Summer, Autumn, and Winter: An Essay principally concerning natural phenomena admitting of interpretation by Chemical Science, and illustrating passages of Scripture.* By Thomas Griffiths, Professor of Chemistry in the Medical College of St. Bartholomew's Hospital; Author of "Recreations in Chemistry," and "Chemistry of the Four Elements." 12mo. pp. 451. Lea & Blanchard, Philadelphia, 1846.

This Essay is founded upon Lectures delivered by the Author at Scientific Institutions in London and Liverpool. It was very recently published in London, and is now republished by the well known Philadelphia house above named. It will be perceived from the title page of the work, that it is not designed so much for the professional as the general reader; and yet we venture to express the opinion that no one can read it, not even the professed Chemist, without both pleasure and profit. We have found it, indeed, one of the most interesting *popular* treatises that we have met with for a long time, on any subject—interesting for the facts which it contains, the inferences and reflections arising from the consideration of these facts, the chaste but glowing language in which it is written, and the beauty and aptness of the illustrations employed by the author to enforce and explain his views. We subjoin one or two brief extracts, not for any thing new that they contain, but to afford to the reader some idea of the kind of matter of which the essay is composed, and the manner in which the subjects are treated.

"Dark soils absorb heat more powerfully, and radiate heat more energetically than light colored soils, which reflect a great portion of heat; we have now to examine how these facts, and others concerning the mere alteration of surfaces in affecting both absorption and radiation of heat, are applicable to the explanation of a beautiful phenomenon of Summer and Autumn.

"The night has been serene, the moon and stars have shed their brilliant light, no clouds have obscured the heavens, no rain has fallen, and yet when we step forth at daybreak, we find the grass and flowers of the field loaded with myriads of drops of water, sparkling like gems in the golden rays of the rising sun.

"We recognize this beautiful phenomenon as Dew;—but from whence has it silently journeyed and arrived during the hours of night?—can the chemist reply?

"He can; and the reply will furnish another example of the power and goodness of God, for 'His favour is as dew upon the grass;' another proof of the ever-watchful care of Him with whom 'the darkness and the light are both alike,' whose hand is equally extended for the protection of the animated creation during its noontide activity and its midnight repose.

"Throughout the fervent glow of a Summer or Autumnal day, the solid opaque earth absorbs heat: this abides upon its mere surface, and is not conducted beneath; but at sunset, if the sky be cloudless and calm, the earth immediately radiates part of the heat upward, and soon becomes many degrees colder than the air directly incumbent upon its surface; accordingly the watery vapor that is present in the yet warm air, is chilled or condensed into drops of water, and these 'distil as the dew' upon the earth, for the refreshment of its productions.

"This phenomenon cannot fail of appearing remarkable, even to the most careless observer, and it becomes yet more so when accurately investigated by the chemist. Examine a garden immediately after sunrise at this season; probably the grass-plat is saturated with dew; the gravel walk is nearly dry; the leaves of the hollyhock are dripping with water; the leaves of the laurel are free from moisture; but all these objects were similarly exposed to the night air, and if dew were a fine rain, as some persons imagine it to be, all should be equally covered with its drops; why is this difference observed?

"Because the surfaces of the various objects differ in their radiating power; the grass-plat and the leaves of the hollyhock are excellent radiators; they throw off heat with great energy, and so becoming cold, they induce a more copious deposition of water from the air than the gravel-walk and the laurel leaves, which, being bad radiators, retain heat and remain so warm that the watery vapor in the air wafts over their surfaces without being chilled or condensed, and therefore they are free from dew.

"Rough or woolly leaves, like the painted or sanded surface of the tin-plate, radiate heat most rapidly, whilst smooth or varnished leaves, like the polished or bright surface of the tin-plate, do not radiate with such energy, and as a consequence of this, the former leaves ensure a more plentiful deposition of dew than the latter.

"From the limits of the garden, let us carry forth these observations and facts into the boundless fields of Nature, and discover the miraculous workings of Providence.

"Barren rocks and soils, by reason of their peculiar hard and compact structure, have neither the power of absorbing nor of radiating heat with great energy; they do not speedily acquire a low temperature during the clear nights of Summer and Autumn.

and as a consequence, dew is scarcely, certainly not abundantly, deposited upon them; it is not required for their support, and they have no vegetable life, or but little of the lowest grade to maintain."

We have given more space to this little work than we are accustomed to bestow on such as are not strictly professional in their character, because it treats of many topics on which physicians, and particularly country physicians, ought to be well informed. If treatises on all the subjects of science, and especially medical science, written in the same familiar style, were generally circulated, it would do more to put down quackery and imposture, by enlightening the minds of the public, than all the conventions and legal enactments in the world.

---

*New Remedies.* By Robley Dunglison, M. D., Professor of the Institutes of Medicine, etc., in the Jefferson Medical College of Philadelphia. *Fifth edition, with extensive additions.* 8vo. pp. 639. Philadelphia, Lea & Blanchard, 1846.

"The enlarged experience of observers," says the author in his preface to this edition, "since the appearance of the fourth edition of this work, has tested the value of many articles contained in it, and given occasion to the incorporation of much new matter with the text. New remedies have also been proposed in no small number; so that the present edition is much larger, and describes many more articles than the last."

A work like this is obviously not suitable for either critical or analytical review. It is, so far as it goes, a dispensatory, in which an account is given of the chemical and physical properties of all the articles recently added to the materia medica and their preparations, with a notice of the diseases for which they are prescribed, the doses, mode of administration, &c. For the accuracy of these statements, those on whose authority they are given are alone responsible, and the names of such are invariably given by the author. Further experience, in some instances, will doubtless correct or limit the over confident opinions of those by whom some of these new remedies are proposed, whilst in other cases, perhaps, remedial virtues will be discovered which they are not now thought to possess. The same remarks are applicable to some of the articles long used, and now proposed for new objects: time and further observation must determine the degree of confidence to which they are entitled. All that can be expected in a work like the present is, that it shall be full in its contents, accurate in its details, and faithful in its references. That the present publication is of this character, need scarcely be said, when we regard the source from whence it comes. No man living, probably, is better acquainted with whatever is new or valuable in medicine than the author.

---

*Special Anatomy and Histology.* By William E. Horner, M. D., Professor of Anatomy in the University of Pennsylvania, Member of the Imperial Medico-Chirurgical Academy of St. Petersburg, of the American Philosophical Society, etc. In two volumes, 8vo. *Seventh edition, with numerous Illustrations.* Lea & Blanchard, Philadelphia, 1846.

The first edition of this work appeared in 1826, previous to which mere *Descriptive Anatomy*, with very little of *General Anatomy* or *Histology*, occupied the pages of works devoted to this subject. A closer investigation of the tissues which make up the various organs and portions of the body, facilitated and in a great degree prompted by improvements in the microscope, has enlarged the boundaries of the science, and added new incentives to the study of this branch of medical knowledge; hence, instead of *one* volume, the work of Professor Horner has expanded into *two*, of portly dimensions.

In a somewhat lengthy *Preface* to the present edition, the author observes:

"In presenting to the profession a seventh edition of his work on Special Anatomy and Histology, the Author remarks, that it is not a mere re-print of the last edition, published three years ago, which itself contained copious additions over its predecessors; but that it has undergone several modifications, and many extensions, derived from the progressive state of the Science of Anatomy."

This edition likewise contains additional illustrations, derived from the best authorities; "and it is placed in a more immediate relation with the volume of plates, by Dr. H. H. Smith, called Anatomical Atlas; they having been selected expressly as an elucidation of its text. This connection has been done by specific references at the foot of the page to the plates in question." So says the *Preface.*

When a work which is purely scientific, and without the advantages of a very correct diction, passes to a seventh edition, as in the present instance, it is pretty conclusive evidence of its merits.

---

*A Manual of Examinations upon Anatomy and Physiology, Surgery, Practice of Medicine, Chemistry, Obstetrics, Materia Medica, Pharmacy, and Therapeutics; to which is added, a Medical Formulary. Designed for Students of Medicine throughout the United States.* By J. L. Ludlow, A.M. M. D. *Second edition, revised and enlarged.* 12mo. pp. 689. Ed. Barrington & George D. Haswell. Philadelphia, 1846.

"Our object is," says the author, "simply, to give at a glance the principal points necessary to guide the student in the prosecution of his studies, and to revive his recollection of subjects treated upon in more voluminous works."

"In this edition, many additions have been made to keep up to the Science as it has advanced, and is advancing; and in some instances a new arrangement of subjects has been deemed advisable."

---

*A System of Anatomy for the use of Students of Medicine.* By Caspar Wistar, M. D., late Professor of Anatomy in the University of Pennsylvania. *With notes and additions,* by William E. Horner, M. D., Professor of Anatomy in the University of Pennsylvania. *Ninth Edition; entirely remodeled, and illustrated by more than one hundred Engravings.* By Joseph Pancoast, M. D., Professor of General, Descriptive and Surgical Anatomy in Jefferson Medical College of Philadelphia, Lecturer on Clinical Surgery, Fellow of the Philadelphia College of Physicians, etc. In two vols. 8vo. Thomas, Cowperthwaite & Co. Philadelphia.

A work which comprises the views and observations of three distinguished teachers of Anatomy, presents more than ordinary claims to the attention of practitioners and students of medicine. The present work, however, has been so long and so extensively before the profession that its merits are too well known to require that we should do more than mention what changes and additions have been made in the present edition.

The text we perceive has undergone revision, and considerable new matter, relating more particularly to histology, has been introduced. Several wood cuts have also been added, and the beautiful copper plate engravings of the arteries by Sir Charles Bell, properly coloured, are inserted, and add very much to the value of the present over former editions.

---

*An Anatomical Description of the Diseases of the Organs of Circulation and Respiration.* By CHARLES EWALD HASSE, M. D., etc. *Translated and Edited* by W. E. SWAINE, M. D., etc. 8vo. pp. 377. Lea & Blanchard, Philadelphia, 1846.

In a former number (August, 1846) we noticed this work of Dr. Hasse in terms of suitable commendation. The fact of its being translated and published under the direction of the Sydenham Society, is alone sufficient evidence of its high character, and our townsmen have conferred a favour on the profession in this country by its re-publication. It is a standard work, and ought to be in the library of every physician.

